# Effect of maternal dietary patterns on infant growth in Baotou, China

**DOI:** 10.1371/journal.pone.0328810

**Published:** 2025-09-04

**Authors:** Jingyu Yang, Hailong Zhang, Mingming Han, Haiying He, Lin Xie

**Affiliations:** 1 Department of Nutrition and Food Hygiene, School of Public Health, Jilin University, Changchun, China; 2 Department of Gynaecology and Obstetrics, The Third Hospital of Baogang Group, Baotou, China; 3 Department of Radiology, The Third Hospital of Baogang Group, Baotou, China; 4 Department of Pediatrics, The Third Hospital of Baogang Group, Baotou, China; Mizan-Tepi University, ETHIOPIA

## Abstract

**Background:**

Maternal dietary patterns (DPs) during pregnancy play a crucial role in fetal development and child growth. This study aims to investigate the association between maternal DPs and infant growth outcomes.

**Methods:**

A prospective cohort study was conducted at The Third Hospital of Baogang Group from January 2020 to January 2022, enrolling 201 mother-child pairs. Maternal dietary intake during pregnancy was assessed, and DPs were identified using principal component analysis. Univariate analysis was performed to determine factors influencing maternal DPs. Infant weight and length were measured at birth and 12 months of age, and growth indicators including weight-for-age z-score (WAZ), height-for-age z-score (HAZ), and body mass index-for-age z-score (BMI Z) were calculated. The impact of maternal DPs on infant growth was analyzed by comparing growth outcomes across different DPs.

**Results:**

Four distinct maternal DPs were identified: FMDP (high intake of fruits, milk, dairy products, and poultry), VBAP (vegetables, beans, algae, and pork), MP (marine products), and TE (tubers and eggs). Maternal education level was a significant factor influencing DP (P < 0.05). Higher adherence to FMDP and MP was associated with greater infant birth weight (P < 0.05), while VBAP was positively associated with birth weight (P < 0.05). Conversely, TE was linked to lower birth weight (P < 0.05). Additionally, FMDP was positively correlated with WAZ and HAZ at birth (P < 0.05), while MP was associated with higher WAZ at birth (P < 0.05). VBAP was positively associated with BMI Z at 12 months (P < 0.05), whereas TE was negatively associated with WAZ at birth (P < 0.05).

**Conclusion:**

Maternal DPs during pregnancy significantly influence infant birth weight, length, and subsequent growth trajectories. These findings highlight the importance of maternal nutrition in offspring early-life growth and development.

## Introduction

It is well-known that nutritional status is associated with chronic diseases, such as chronic degenerative disorders [1], obesity [2], cardiovascular diseases [2], and chronic kidney disease [3]. Maternal nutrition during pregnancy represents a foundational determinant of developmental programming, with substantial implications for offspring metabolic health including obesity susceptibility, cardiometabolic dysfunction, and diabetes risk [4]. As fetal nutrient supply is entirely dependent on maternal reserves, the impact of gestational nutritional status on infant developmental outcomes has attracted increasing attention from scholars. Previous studies have shown a dual nature of nutritional influence on infant growth [4,5]. While adequate micronutrient and macronutrient intake during pregnancy supports optimal fetal growth and reduces chronic disease predisposition [5], excessive caloric intake elevates risks of macrosomia, childhood obesity, and subsequent metabolic syndrome [4]. This dichotomy underscores the imperative to establish evidence-based gestational nutritional guidelines for pediatric health promotion.

Regional dietary patterns (DP) in China demonstrate remarkable heterogeneity influenced by cultural traditions, socioeconomic factors, and geographical constraints [6–8]. Baotou, an industrial city in Inner Mongolia Autonomous Region, presents a unique nutritional landscape shaped by Mongolian-Han cultural integration. The local cuisine features predominant consumption of red meats (especially lamb and beef), traditional preparations including grilled meats and hotpot, and culturally significant dairy products like milk tea and yogurt. Limited vegetable diversity, primarily root vegetables (potatoes, onions, carrots) typically stewed with meats, reflects climatic adaptations. While modernization has introduced Western fast food and external culinary influences, persistent dietary characteristics coupled with socioeconomic stratification between industrial and rural populations create a compelling context for nutritional epidemiology research.

Operationalized through statistical methods such as principal component analysis (PCA), DPs represent clustered food consumption behaviors [9]. However, DP characterization remains methodologically inconsistent across studies due to differences in dietary assessment tools, population-specific food availability, and cultural determinants. These variations can affect how DPs are linked to infant growth outcomes, particularly given the mediating roles of nutrient intake, food availability, and maternal health status. Standardization of DP assessment is therefore essential for accurately elucidating gestational nutritional impacts on infant physical growth.

At present, emerging literature has focused on the associations between gestational DPs and infant growth. Knudsen et al. [10] and John et al. [11] suggested that a DP in pregnancy dominated by red meats, processed meats, and high-fat diet increased the risk of small for gestational age (SGA). A study further links suboptimal diet quality during pregnancy to preterm birth [12], while evidence correlates specific DPs with birth metrics including weight, length, head circumference, and age-adjusted Z-scores [weight-for-age (WAZ), height-for-age (HAZ), body mass index (BMI)-for-age (BMI Z) [13,14]. Although the effect of maternal DPs during pregnancy was extensively researched, current research predominantly examines short-term perinatal outcomes, with limited longitudinal data on childhood growth trajectories. A study from the United States found associations between maternal DPs during pregnancy and BMI Z, overweight, and obesity in infants aged 1–3 years, which were statistically non-significant after adjusting for confounding factors [15]. Similarly, van den Broek M also reported that maternal DPs during pregnancy did not affect the BMI of infants [16]. Interestingly, a Singaporean study revealed that maternal DPs in pregnancy could affect BMI Z-scores of infants beyond 18 months after correcting for confounding factors [16]. These contradictory findings highlight critical knowledge gaps regarding the temporal persistence of gestational nutritional programming effects.

Given this unresolved scientific controversy, this study aims to systematically investigate the longitudinal associations between maternal DPs during pregnancy and infant growth trajectories in a cohort from northern China.

## Materials and methods

### Study participants and design

The present study utilized data from a prospective maternal and child cohort at The Third Hospital of Baogang Group from January 2020 to January 2022. Recruitment targeted pregnant women attending antenatal clinics in their third trimester (≥ 28 weeks of gestation), with baseline assessments including maternal pre-pregnancy BMI, blood pressure, and medical history. Nutritional assessment was conducted using the Food Frequency Questionnaire (FFQ) to quantify dietary intake. Lifestyle factors such as physical activity and socioeconomic status were also recorded. Mother-child pairs were followed up at delivery and one year postpartum to examine the associations between maternal DPs and infant growth parameters. The study adhered to the Declaration of Helsinki, and was approved by the Ethics Committee of The Third Hospital of Baogang Group. Written informed consent was obtained from all participants.

A total of 253 pregnant women were initially recruited. After applying the inclusion and exclusion criteria, 201 eligible mother-child pairs were included in the final analysis. Inclusion criteria were as follows: ① maternal age 18–35 years; ② singleton pregnancy; ③ full-term delivery (gestational age ≥ 37 weeks); and ④ infant birth weight ≥2,500 g. Exclusion criteria were: ① history of smoking or alcohol consumption during pregnancy; ② pre-existing chronic diseases (including hypertension, diabetes, thyroid disorders, mental illness, or pregnancy complications); ③ infectious diseases (such as chronic hepatitis B, syphilis, acquired immune deficiency syndrome); and ④ missing data on sociodemographic variables, anthropometrics (weight/height), or FFQ. These criteria were applied to minimize confounding factors that could influence fetal growth or dietary reporting, thus improving the homogeneity and reliability of the study population.

Among the initial 253 women, 21 failed to meet the inclusion criteria, and 31 were excluded due to missing or incomplete FFQ or anthropometric data.

### Dietary assessment

A dietary survey was conducted using a 3-day 24-hour retrospective method [17]. The food was categorized into 14 groups, including Rice, Fruits, Milk and dairy products, Poultry, Vegetables, Beans and nuts, Algae, Pork, Marine products, Coarse cereals, Soups, Flour products, Tubers, and Eggs, as described in previous literature [18]. Dietary food consumption patterns before pregnancy were assessed by an FFQ [19], consisting of 50 food items. The FFQ implementation revealed 31 incomplete responses, primarily in categories such as Dairy products, Beans and nuts, and Marine products, which were handled by multiple imputation using chained equations (MICE), a validated approach for minimizing bias in DP derivation when handling missing dietary data. To reduce recall bias, investigators provided a unified food model and food images during the survey, which facilitated the respondents to recall their daily food intake. Given the documented temporal stability of DPs throughout pregnancy [20,21], the present study used late-pregnancy DP analysis to represent whole-pregnancy nutritional exposure. DPs were defined as predominant food group combinations derived from factor loadings (> 0.3) via PCA. For example, FMDP emphasized nutrient-dense foods (fruits, dairy products), whereas TE reflected energy-dense, low-variety diets.

### Outcome assessment

Infant birth weight (g) and length (cm) at birth and 1 year old were extracted from the medical records of the The Third Hospital of Baogang Group. All measurements were performed by trained nursing staff using calibrated equipment. At both time points, infant weight was measured to the nearest 0.01 kg using electronic infant scales, and length/height was measured to the nearest 0.1 cm using a standard stadiometer or infantometer. Measurement protocols were consistent across timepoints to ensure comparability. Following the World Health Organization (WHO) Child Growth Standards [22], age- and sex-adjusted Z-scores were calculated for critical growth parameters, including WAZ, HAZ, and BMI Z.

### Basic demographic information collection

Basic demographic information was collected via a structured questionnaire, including maternal age, education level, pre-pregnancy weight, height, and family economic status. In addition, after delivery, the data on delivery method, obstetric complications, bleeding volume, neonatal outcomes were collected.

### Power analysis

Using one-way analysis of variance (ANOVA) as the primary analysis for comparing infant growth indicators (e.g., birth weight, birth length, WAZ, HAZ, BMI Z) among four DP groups (FMDP, VBAP, MP, and TE), we assumed a medium effect size (Cohen’s f = 0.25), a significance level of α = 0.05, and a desired power (1-β) of 80%. Based on these parameters, power analysis (using G*Power 3.9 software) indicated a minimum required sample size of approximately 180 participants (i.e., about 45 subjects per group).

### Statistical analysis

Data entry was performed using EpiData 3.0 by two independent staff members, with cross-validation for accuracy. Missing data were handled as described above. Dietary nutrient intakes were calculated using Golden Key Maternal Nutrition software (Wincome, Shanghai) and the China Food Composition Table 2009. In the analysis of DPs, 15 food groups were extracted using PCA, with Kaiser-normalized varimax rotation. The Kaiser-Meyer-Olkin (KMO) greater than 0.6 and the P-value of the Bartlett’s sphericity test less than 0.05 were required to ensure that the sample was suitable for PCA. In statistical analyses, we considered possible confounding factors, including maternal age, education level, family economic status, pre-pregnancy weight, etc., and included them in the model as covariates. For the measurement data, we used mean ± standard deviation (SD) to represent the normally distributed data and the median (P25, P75) to represent the skewed data. Differences in continuous variables (such as birth weight, length, and growth indicators) among the four DP groups were assessed using one-way ANOVA. When a significant overall difference was detected, Bonferroni post hoc tests were performed to identify pairwise differences. Disaggregated data were expressed as n (%), and the differences between the groups were analyzed by a Chi-square test. Spearman correlation analysis was used to evaluate the correlations between DPs and infant growth indicators. All statistical tests were two-tailed, and P < 0.05 was considered significant. All analyses were performed using SPSS Statistics software (version 16.0; IBM Corporation).

## Results

### Basic information of pregnant women and infants

The median age of pregnant women was 26.0 years, with 40.8% having ≤ 9 years of education. In terms of annual household income per capita, 39.3% earned less than 10,000 yuan. The median height was 158.5 cm, and the median pre-pregnancy weight was 51.2 kg, with a median BMI of 19.1 kg/m^2^. Delivery method comparison showed that 52.2% underwent cesarean sections, while 47.8% had vaginal deliveries. For infants, 51.2% were male and 48.8% were female. The median gestational age at delivery was 40.0 weeks, with a median birth weight of 3.3 kg and a median birth height of 48.6 cm. This data provide a snapshot of the demographic and health characteristics of the mothers and their newborns in the study area, as detailed in Table 1.

**Table 1:** Basic information of pregnant women and infants.

Characteristics	Number/Medium/Mean	Ratio (n%)/(P25, P75)/SD
Pregnant women		
Age (years)	26.0	(23.0,29.0)
Education level (years)		
≤ 9 years	82	40.8%
9 years< time ≤ 12 years	67	33.3%
> 12 year	52	25.9%
Annual household income per capita (yuan)		
< 10000 yuan	79	39.3%
10000 yuan-30000 yuan	63	31.3%
30000 yuan-50000 yuan	27	13.4%
>50000 yuan	32	15.9%
Height (cm)	158.5	(156.7,160.4)
Pre-pregnancy weight (kg)	51.2	(47.5,53.9)
Pre-pregnancy BMI (Kg/m2)	19.1	(20.2,21.4)
< 18.5	28	13.9%
18.5-23.9	167	83.1%
≥ 24	16	7.96%
Delivery method		
Cesarean section	105	52.2%
Vaginal delivery	96	47.8%
Gravidity (time)		
Once	124	61.7%
Twice	57	28.4%
≥ Three times	20	10.0%
Parity (time)		
Once	144	71.6%
Twice	55	27.4%
≥ Three times	2	1.00%
Infants		
Gender		
Male	103	51.2%
Female	98	48.8%
Gestational age (week)	40.0	(38.0,41.0)
Birth weight (kg)	3.3	(2.9,3.5)
	3.26	0.43
Birth height (cm)	48.6	(45.4,51.4)
	48.57	3.89

### Infant growth trajectories

**Fig 1:**
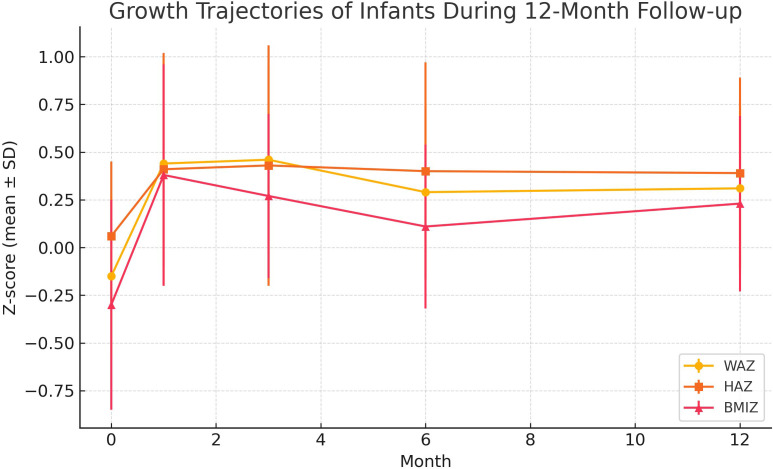
Growth trajectories of infants during 12-month follow-up. Note: Changes in weight-for-age Z-score (WAZ), height-for-age Z-score (HAZ), and BMI-for-age Z-score (BMI Z) from birth to 12 months. Values are presented as mean ± standard deviation.

As shown in **Table 2** and **Fig 1**, the WAZ, HAZ, and BMI Z were −0.15 ± 0.43, 0.06 ± 0.39, and −0.30 ± 0.55, respectively, at birth. At 12 months, the WAZ, HAZ, and BMI Z were 0.31 ± 0.45, 0.39 ± 0.50, and 0.23 ± 0.46, respectively. The growth of infants plateaued at 6 months, as indicated by the stabilization of WAZ and HAZ between 6 and 12 months, while absolute weight and length continued to increase. All growth indicators were higher than the WHO standards.

**Table 2:** Infant growth status during 12-month follow-up.

Months	WAZ	HAZ	BMI Z
0	−0.15 ± 0.43	0.06 ± 0.39	−0.30 ± 0.55
1	0.44 ± 0.54	0.41 ± 0.61	0.38 ± 0.58
3	0.46 ± 0.49	0.43 ± 0.63	0.27 ± 0.43
6	0.29 ± 0.45	0.40 ± 0.57	0.11 ± 0.43
12	0.31 ± 0.45	0.39 ± 0.50	0.23 ± 0.46

**Note:** WAZ: Weight-for-age Z-score; HAZ: Height-for-age Z-score; BMI Z: Body mass index-for-age Z-score.

### PCA and multicollinearity diagnostics for DP variables

PCA of the 14 food groups identified four components with eigenvalues greater than 1.0, cumulatively explaining 60.1% of the total dietary variance. All variance inflation factor (VIF) values for the included variables were below 2.0, indicating no significant multicollinearity among the food groups, as shown in **Table 3**.

**Table 3:** Principal component analysis and multicollinearity diagnostics for dietary pattern variables.

Component	Eigenvalue	Variance Explained (%)	Cumulative Variance (%)	VIF
Rice	2.789	19.919	19.919	
Fruits	2.216	15.83	35.75	1.668
Milk and dairy products	2.06	14.711	50.461	1.438
Poultry	1.35	9.642	60.104	1.479
Vegetables	0.844	6.031	66.135	1.54
Beans and nuts	0.726	5.188	71.323	1.577
Algae	0.669	4.778	76.101	1.595
Pork	0.598	4.27	80.37	1.422
Marine products	0.571	4.075	84.446	1.563
Coarse cereals	0.518	3.701	88.146	1.578
Soups	0.495	3.538	91.684	1.434
Flour products	0.452	3.228	94.912	1.601
Tubers	0.386	2.755	97.668	1.244
Eggs	0.327	2.332	100	1.192

### DP division

**Fig 2:**
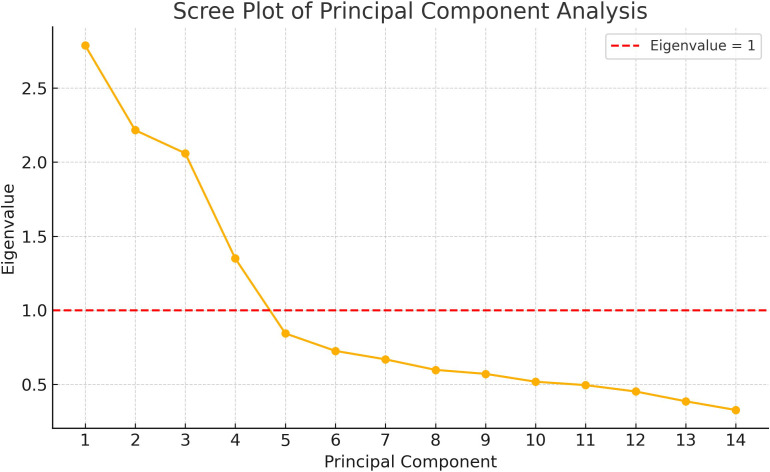
Scree plot from principal component analysis (PCA) of dietary patterns. Note: The plot shows the eigenvalues for each principal component. The red dashed line indicates an eigenvalue of 1, which is the threshold commonly used to determine the number of components to retain.

Following varimax rotation in PCA, four distinct DPs were derived based on food groups with factor loadings > 0.3. The four DPs were summarized as follows: ① Pattern 1 (mainly fruits, milk and dairy products, and poultry, FMDP); ② Pattern 2 (mainly vegetables, beans, algae, and pork, VBAP); ③ Pattern 3 (mainly marine products, MP); ④ Pattern 4 (mainly tubers and eggs, TE), as illustrated in **Table 4** and **Fig 2**.

**Table 4:** Rotated component matrix among different dietary patterns.

Food group	Pattern 1	Pattern 2	Pattern 3	Pattern 4
Rice	0.776			
Fruits	0.818			
Milk and dairy products	0.741			
Poultry	0.758			
Vegetables		0.730		
Beans and nuts		0.756		
Algae		0.758		
Pork		0.718		
Marine products			0.757	
Coarse cereals			0.709	
Soups			0.766	
Flour products			0.757	
Tubers				0.793
Eggs				0.797

**Note:** Only food groups with absolute factor loadings > 0.3 are listed in the table;

Extraction method: Principal component analysis;

Rotation method: Orthogonal varimax rotation with Kaiser normalization.

### Maternal characteristics among four DPs

Factor scores were calculated for the four DPs, and the maximum factor score was determined as the DP of each pregnant woman. The distribution of the four DPs was as follows: 49 pregnant women in the FMDP, accounting for 24.4%; 54 in the VBAP, accounting for 26.9%; 52 in the MP, accounting for 25.9%; 46 in the TE, accounting for 22.9%. The population characteristics of the four DPs are shown in **Table 5**. There were no significant differences in maternal age, annual household income per capita, pre-pregnancy BMI, or gravidity among the four DPs (P > 0.05). However, education level among the four groups exhibited significant differences (P < 0.05).

**Table 5:** Maternal demographics by different dietary patterns.

Characteristics	FMDP(n = 49)	VBAP(n = 54)	MP(n = 52)	TE(n = 46)	P value
Age	25.7 ± 4.3	26.7 ± 4.8	25.4 ± 4.6	26.9 ± 3.9	0.253
Education level (years)					
≤9 years	27 (23.5%)	18 (33.3%)	27 (48.2%)	10 (21.7%)	
9 years<time≤12 years	13 (35.3%)	17 (31.48%)	17 (30.4%)	20 (43.5%)	
>12 year	9 (41.2%)	19 (35.2%)	12 (21.4%)	16 (34.8%)	0.022
Annual household income per capita					
< 10000 yuan	17 (34.7%)	23 (42.6%)	19 (36.5%)	20 (43.5%)	
10000 yuan- 30000 yuan	20 (40.8%)	11 (20.4%)	16 (30.8%)	16 (34.8%)	
30000 yuan −50000 yuan	4 (8.2%)	9 (16.7%)	9 (17.3%)	5 (10.9%)	
> 50000 yuan	8 (16.3%)	11 (20.4%)	8 (15.4%)	5 (10.9%)	0.530
Pre-pregnancy BMI					
<18.5	3 (6.1%)	7 (13.0%)	5 (9.6%)	11 (23.9%)	
18.5-23.9	41 (83.7%)	44 (81.5%)	44 (84.6%)	30 (65.2%)	
≥24	5 (10.2%)	3 (5.6%)	3 (5.8%)	5 (10.9%)	0.148
Parity					
Once	39 (65.5%)	34 (63.0%)	29 (55.8%)	31 (67.4%)	
≥twice	10 (34.6%)	20 (37.0%)	23 (44.2%)	15 (32.6%)	0.081

**Note:** FMDP: Fruits, milk, dairy products, and poultry pattern; VBAP: Vegetables, beans, algae, and pork pattern; MP: Marine products pattern; TE: Tubers and eggs pattern; SD: Standard deviation.

### DP associations with infant weight and length

**Table 6** presents birth weight and length data across four groups (FMDP, VBAP, MP, TE) and three quartiles (Q1, Q2, Q3). Significant differences were found in birth weight and length for the MP and TE groups, with lower values in Q3 compared to Q1. The VBAP group showed significantly greater birth length in Q2 and Q3 compared to Q1(P < 0.05).

**Table 6:** Variance analysis of association between DP scores and birth weight and length.

	FMDP	VBAP	MP	TE
	Mean±SD	Mean±SD	Mean±SD	Mean±SD
Birth weight				
Q1	3.1 ± 0.5	3.2 ± 0.5	3.2 ± 0.4	3.4 ± 0.2
Q2	3.4 ± 0.4	3.3 ± 0.3	3.2 ± 0.4a	3.3 ± 0.3
Q3	3.4 ± 0.4a	3.3 ± 0.4a	3.5 ± 0.4a	3.0 ± 0.5a
Birth length				
Q1	44.3 ± 4.2	45.5 ± 3.8	43.2 ± 4.6	47.1 ± 4.4
Q2	46.3 ± 4.5	46.8 ± 2.7	46.0 ± 4.6a	46.3 ± 3.6
Q3	46.4 ± 3.8	48.0 ± 3.9a	47.5 ± 3.6	44.6 ± 4.0

**Note:** Differences among groups were assessed by ANOVA followed by Bonferroni post hoc test. a, compared to Q1, P < 0.05; b, compared to Q2, P < 0.05.

### Longitudinal associations between DPs and infant growth

Correlation analysis showed that FMDP was positively associated with WAZ and HAZ of infants at birth (P < 0.05). MP was positively associated with WAZ of infants at birth (P < 0.05). VBAP was positively associated with BMI Z of infants at 1 year old (P < 0.05). In addition, TE was negatively associated with WAZ of infants at birth (P < 0.05), as seen in the **Table 7**.

**Table 7:** Relation of DP scores and WAZ, HAZ, and BMI Z of offspring at 1 year.

	Months	FMDP		VBAP		MP		TE	
		r	P	r	P	r	P	r	P
WAZ	0	0.586	<0.001*	0.185	0.179	0.435	0.001*	−0.410	0.005*
	12	0.232	0.109	0.356	0.008	0.196	0.163	0.223	0.136
HAZ	0	0.438	0.002*	0.056	0.688	0.025	0.858	0.150	0.318
	12	0.229	0.114	0.197	0.154	0.131	0.355	−0.005	0.974
BMI Z	0	0.045	0.758	0.215	0.119	0.340	0.014	−0.080	0.599
	12	0.063	0.668	0.350	0.010*	0.257	0.066	0.115	0.447

**Note:** Bonferroni correction was applied for multiple comparisons among dietary patterns for each outcome variable (corrected significance level: P < 0.0125). Significant correlations after correction are marked with *.

## Discussion

Our longitudinal analysis demonstrates that distinct maternal DPs during late pregnancy are significantly associated with both short-term and long-term growth outcomes in infants. Specifically, FMDP and MP were associated with higher birth weight and length, while TE was linked to lower birth weight. Additionally, adherence to VBAP was positively correlated with improved BMI Z at one year of age.

Our PCA identified four distinct PDs, including FMDP, VBAP, MP, and TE, demonstrating conceptual alignment with a previous study [10]. Yang J [22] and Zhang H [23] reported a vegetarian type (potatoes, grains, vegetables) and animal-based food type (blood, viscera, salted eggs, meat products) during pregnancy, respectively. Scholars from Denmark found three DPs, named the Western model (mainly red meats, processed meats, and high-fat milk), the Health model (mainly vegetables, fruits, and fish), and the intermediate model [10]. However, a study from Finland proposed seven DPs [24]. Although different studies have different classifications and nomenclature, there are also certain similarities. It is worth mentioning that DP during pregnancy is difficult to unify due to the differences in regions, races, and population composition. In addition, some researchers suggest that pregnant woman with higher education levels tend to choose healthier DPs [25,26]. The present study also observed an educational gradient in VBAP adherence, corroborating global trends linking maternal education with nutrient-dense food selection [25]. This association likely reflects health literacy-mediated dietary decision-making, where educated mothers prioritize foods with high nutrient density. Contrary to expectations, there were no significant differences in other sociodemographic factors, suggesting that DP determination operates through knowledge-based pathways rather than purely economic constraints.

Our findings also delineate specific nutrient-mediated pathways through which maternal DPs during pregnancy influence fetal birth weight and length. It was found that FMDP and MP increased birth weight and length, VBAP increased birth weight, and TE decreased birth weight and length, which are roughly consistent with the existing conclusions. The food in VBAP and FMDP contains many proteins and nutrients, which is consistent with the model of “high nutrition”, “high protein” and “health consciousness”. In addition, MP contains more seafood compared to other dietary models, in line with the “Mediterranean model” [27]. Conversely, TE exhibited suboptimal nutrient density, mirroring traditional starch-centric dietary models characterized by high glycemic load and limited micronutrient diversity. Previous studies have shown that iron and folic acid in nutrients, fruits, vegetables, low-fat milk, and lean meats are beneficial for an appropriate birth weight [28,29]. In addition, the present study found that FMDP was positively associated with WAZ and HAZ of infants at birth, MP was positively associated with WAZ of infants at birth, and TE was negatively associated with WAZ of infants at birth. These results substantiate those in birth weight and length, indicating that maternal DPs during pregnancy have an impact on growth at birth. Emerging evidence further demonstrates that maternal DPs may influence infant growth not only through direct nutritional effects but also via epigenetic mechanisms that regulate gene expression during critical periods of development [30–32]. For instance, Boker et al. [30] and Tozzi et al. [33] reported significant associations between prenatal nutrition, DNA methylation, and early childhood growth outcomes. These findings align with our observations and suggest a complex interplay between maternal diet and offspring health.

Our findings extend the developmental origins of health and disease paradigm by demonstrating enduring nutritional programming effects beyond the perinatal period. Many studies have shown that the impact of DPs during pregnancy on offspring is limited to birth outcomes [34,35], and their long-term effects are relatively rare. Our study explored the impact of maternal DPs during pregnancy on the growth of infants at one year old, revealing that VBAP was positively associated with BMI Z. This temporal persistence aligns with a previous study documenting DP effects beyond 18 months [36], yet contrasts with null findings in Western populations. The discrepancies potentially attributable to: firstly, the correction factors are not the same, secondly, the naming of DPs is not completely consistent, and thirdly, the use of regression statistical methods can only obtain direct effects. Hence, VBAP has an impact on the long-term growth of offspring (at 1 year). Compared to other DPs, the VBAP mainly includes vegetables, beans, algae, and pork, demonstrating the highest protein content. Previous studies have already suggested that high-quality protein is an important nutrient for promoting children’s growth [37,38]. In contrast, the TE is relatively unfavorable for growth due to its dual burden of low nutrient density and suboptimal energy provision. It can be seen that the impact of DPs on the growth and development of offspring is diverse, with positive and negative effects, direct and indirect effects, and different effects on the early and late stages. In summary, this study demonstrates that DPs during pregnancy have a certain impact on the growth and development of offspring over some time after birth.

The present study still has several limitations. Firstly, although the overall sample size met the requirement of power analysis, certain DP subgroups (e.g., TE and MP) had relatively small participant numbers, potentially reducing statistical power for subgroup comparisons and affecting the generalizability of the findings. Secondly, the study did not adjust for delivery method (cesarean section versus vaginal delivery), which may have introduced residual confounding. Future research should include delivery method as a covariate to better control for its potential impact on infant outcomes. Thirdly, dietary intake was assessed only in late pregnancy and assumed to represent the entire gestational period. While previous studies suggest that DPs remain relatively stable after the first trimester, some early-pregnancy changes may not have been captured. Additionally, as this was a single-center study conducted in a specific region, the findings may not be directly generalizable to other populations. The study is also subject to recall bias due to reliance on the 3-day 24-hour dietary recall, which may have resulted in under- or overestimation of food groups. Moreover, despite adjusting for several major confounders, residual confounding may persist, particularly from unmeasured factors such as maternal physical activity levels and breastfeeding practices, which can also affect infant growth trajectories. Future studies with larger, more diverse cohorts and repeated dietary assessments are needed to further validate and extend these results.

## Conclusion

There are four types of DP during pregnancy, including FMPD (mainly fruits, milk, dairy products, and poultry), VBAP (mainly vegetables, beans, algae, and pork), MP (mainly marine products), and TE (mainly tubers and eggs). In addition, VBAP promotes short-term and long-term growth of infants at 1 year old. However, a further research of expanding sample size is needed.

## Supporting information

S1 Table Data of all pregnant women. (SAV)

S2 Table Data of all infants. (SAV)

S3 Table Correlation analysis between dietary pattern factor scores and Z-scores of infants. (SAV)

S4 Table Z-scores for dietary intake in pregnant women. (SAV)

S5 Table Dietary intake of pregnant women. (SAV)

S6 Table All data summary files. (XLSX)

S7 Table Codebook for all variables used in the study. (SAV)
