# Correction: Effect of maternal dietary patterns on infant growth in Baotou, China

**DOI:** 10.1371/journal.pone.0338114

**Published:** 2025-12-04

**Authors:** Jingyu Yang, Hailong Zhang, Mingming Han, Haiying He, Lin Xie

There are errors in the Funding statement. The correct Funding statement is as follows: This study is supported by Health and Hygiene Research Project of the Metallurgical Safety and Health Branch of the China Metallurgical Society (Project No. jkws202083 to HZ; No. jkws202371 to HZ); Baotou City health science and technology plan project (Project No. wsjkkj084 to HZ; No. 2023wsjkkj88 to HZ); Science and Technology Program of the Joint Fund of Scientific Research for the Public Hospitals of Inner Mongolia Academy of Medical Sciences (Project No. 2023GLLH0252 to HZ).

## References

[1] Yang J, Zhang H, Han M, He H, Xie L. Effect of maternal dietary patterns on infant growth in Baotou, China. PLoS One. 2025;20(9):e0328810. doi: 10.1371/journal.pone.0328810 40906761 PMC12410801

