# Supplementary material for: Effect of maternal dietary patterns on infant growth in Baotou, China
Source: PLoS One. 2025 Sep 4;20(9):e0328810. doi: 10.1371/journal.pone.0328810 (PMC12410801; doi:10.1371/journal.pone.0328810)
Supplement: S7 Table — (SAV) [file pone.0328810.s007.docx]

Supplementary Table 7 Codebook for All Variables Used in the study

| Variable Name | Description | Coding/Unit/Example | Additional Notes / Calculation Method |
| --- | --- | --- | --- |
| **Maternal Information** |  |  |  |
| maternal_id | Unique maternal ID number | Numeric (e.g., 001, 002, ...) |  |
| age | Maternal age at enrollment | Years (e.g., 26) |  |
| education_years | Years of formal education | Integer (e.g., 9, 12, 16) |  |
| income | Annual household income level | 1 = <10k; 2 = 10–30k; 3 = 30–50k; 4 = >50k RMB | See Methods |
| height | Maternal height (pre-pregnancy) | cm (e.g., 160.0) |  |
| weight_pre | Pre-pregnancy weight | kg (e.g., 52.0) |  |
| bmi_pre | Pre-pregnancy BMI | kg/m² (e.g., 20.3) | weight/(height/100)^2 |
| gravidity | Number of pregnancies | Integer (e.g., 2) |  |
| parity | Number of live births | Integer (e.g., 1) |  |
| delivery_method | Delivery mode | 1 = Vaginal, 2 = Cesarean |  |
| **Infant Information** |  |  |  |
| infant_id | Unique infant ID (matches maternal_id) | Numeric |  |
| gender | Infant gender | 1 = Male, 2 = Female |  |
| gestational_age | Gestational age at delivery | Weeks (e.g., 39) |  |
| birth_weight | Birth weight | kg (e.g., 3.35) |  |
| birth_length | Birth length | cm (e.g., 49.5) |  |
| WAZ_0 | Weight-for-age Z-score at birth | Numeric (e.g., 0.15) | WAZ, HAZ, and BMIZ were calculated based on the WHO Child Growth Standards (2006) using the LMS method. Z-scores were computed with WHO Anthro software (https://www.who.int/tools/child-growth-standards/software). |
| HAZ_0 | Height-for-age Z-score at birth | Numeric (e.g., -0.10) | WHO standard, see before |
| BMIZ_0 | BMI-for-age Z-score at birth | Numeric (e.g., -0.30) | WHO standard, see before |
| WAZ_12 | Weight-for-age Z-score at 1 year | Numeric (e.g., 0.21) | WHO standard, see before |
| HAZ_12 | Height-for-age Z-score at 1 year | Numeric (e.g., 0.33) | WHO standard, see before |
| BMIZ_12 | BMI-for-age Z-score at 1 year | Numeric (e.g., 0.12) | WHO standard, see before |
| **Dietary Pattern Scores** |  |  |  |
| FMDP_score | Score for Fruit, Milk, Dairy, and Poultry Pattern | Numeric (e.g., 0.81) | Extracted by PCA, see Methods |
| VBAP_score | Score for Vegetables, Beans, Algae, and Pork Pattern | Numeric (e.g., -0.19) | Extracted by PCA, see Methods |
| MP_score | Score for Marine Products Pattern | Numeric (e.g., 0.26) | Extracted by PCA, see Methods |
| TE_score | Score for Tuber and Egg Pattern | Numeric (e.g., -0.09) | Extracted by PCA, see Methods |
| DP_group | Primary dietary pattern group | 1 = FMDP, 2 = VBAP, 3 = MP, 4 = TE | Determined by highest factor score |
| **FFQ Food Group Intake (grams/day)** |  |  |  |
| Rice | Steamed rice, rice porridge, glutinous rice | g/day |  |
| Fruit | Apple, banana, pear, orange, watermelon, seasonal fruits | g/day |  |
| Milk and dairy | Milk, yogurt, cheese, milk powder | g/day |  |
| Poultry | Chicken, duck, goose | g/day |  |
| Vegetable | Chinese cabbage, spinach, tomato, cucumber, lettuce, broccoli | g/day |  |
| Beans and nuts | Soybeans, tofu, kidney beans, peanuts, walnuts, almonds | g/day |  |
| Algae | Seaweed, kelp, laver | g/day |  |
| Pork | Lean pork, pork belly, ribs | g/day |  |
| Marine products | Fish, shrimp, crab, shellfish, squid | g/day |  |
| Coarse cereals | Millet, corn, sorghum, oats, buckwheat | g/day |  |
| Soup | Vegetable soup, chicken soup, bone soup, fish soup | g/day |  |
| Flour product | Steamed bun, noodles, dumplings, bread, pancakes | g/day |  |
| Tuber | Potato, sweet potato, taro, yam | g/day |  |
| Egg | Chicken egg, duck egg, quail egg | g/day |  |
